# Supplementary material for: A Randomized Study of Nutritional Supplementation in Patients with Unilateral Wet Age-Related Macular Degeneration
Source: Nutrients. 2021 Apr 10;13(4):1253. doi: 10.3390/nu13041253 (PMC8069087; doi:10.3390/nu13041253)
Supplement: Supplementary file 1 [file nutrients-13-01253-s001.pdf]

Article

A Randomized Study of Nutritional Supplementation in Patients with Unilateral Wet Age-related Macular Degeneration

Alfredo García-Layana [1], Sergio Recalde-Maestre [1,*], Maria Hernandez [1], Maximino J. Abraldes [2], João Nascimento [3], Emiliano Hernández-Galilea [4], Begoña Olmedilla-Alonso [5], Jose Juan Escobar-Barranco [6], Miguel Angel Zapata [7], Rufino Silva [8], Mariana Caballero Arredondo [9], M. Carmen Lopez-Sabater [9], Silvia Mendez-Martínez [10], Nieves Pardiñas-Barón [10], Pilar Calvo[10] and Patricia Fernández-Robredo[1]

1 Retinal Pathologies and New Therapies Group, Experimental Ophthalmology Laboratory, Department of Ophthalmology, Clínica Universidad de Navarra, 31008 Pamplona, Spain. Navarra Institute for Health Research, IdiSNA, 31008 Pamplona, Spain. Red Temática de Investigación Cooperativa Sanitaria en Enfermedades Oculares (Oftared), Instituto de Salud Carlos III, Madrid. Spain. aglayana@unav.es, srecalde@unav.es, mahersan@unav.es, pfrobredo@unav.es
2 Department of Ophthalmology, Complexo Hospitalario Universitario de Santiago de Compostela, Universidad de Santiago de Compostela, Santiago de Compostela, Spain. Red Temática de Investigación Cooperativa Sanitaria en Enfermedades Oculares (Oftared), Instituto de Salud Carlos III Madrid, Spain. maxiabraldes@gmail.com
3 Retinal Department, Ophthalmology, Retina Lisbon Institut, Lisbon, Portugal. joaocnascimento@sapo.pt
4 Department of Ophthalmology, University of Salamanca, University Hospital of Salamanca, Salamanca, Spain. egalilea @usal.es
5 Department of Metabolism and Nutrition, Institute of Food Science, Technology and Nutrition (ICTAN-CSIC), Madrid, Spain. BOlmedilla@ictan.csic.es
6 Department of Ophthalmology, Hospital Dos de Maig, Barcelona, Spain. escobarjou@yahoo.es
7 Department of Ophthalmology, Hospital Vall Hebron, Barcelona, Spain. zapatavictori@hotmail.com
8 Faculty of Medicine, Coimbra Institute for Clinical and Biomedical Research (iCBR), University of Coimbra, Coimbra, Portugal; Ophthalmology Department, Centro Hospitalar e Universitário de Coimbra (CHUC), Portugal; Association for Innovation and Biomedical Research on Light and Image (AIBILI), Coimbra, Portugal. rufino.silva@oftalmologia.co.pt
9 Nutrition, Food Science and Gastronomy, University of Barcelona, Barcelona, Spain. mariana.cbllro@gmail.com
10 Department of Ophthalmology, Miguel Servet University Hospital, Zaragoza, Spain; Miguel Servet Ophthalmology Research Group (GIMSO) Aragon Health Research Institute (IIS Aragon), University of Zaragoza, Spain. mclopez@ub.edu; silviamendezmartinez@hotmail.com; npardinasb@yahoo.com; xenatrance@yahoo.es
* Correspondence: srecalde@unav.es; Tel.: +34948255400; Sergio Recalde-Maestre, MD, PhD, Retinal Pathologies and New Therapies Group, Experimental Ophthalmology Laboratory, Department of Ophthalmology, Clínica Universidad de Navarra, Av. de Pío XII, 36, 31008 Pamplona, Spain.

Citation: Lastname, F.; Lastname, F.; Lastname, F. Title. *Nutrients* 2021, *13*, x. https://doi.org/10.3390/xxxxx

Academic Editor: Firstname Lastname
Received: date
Accepted: date
Published: date

Publisher's Note: MDPI stays neutral with regard to jurisdictional claims in published maps and institutional affiliations.

Copyright: © 2021 by the authors. Submitted for possible open access publication under the terms and conditions of the Creative Commons Attribution (CC BY) license (http://creativecommons.org/licenses/by/4.0/).

Supplementary Material

Table S1. Composition of Retilut® (intervention) and Theavit® (control).

Composition (mg*)	Intervention Retilut® (2 tablets)	Control Theavit® (2 tablets)
Vitamin C	160	120
Vitamin E	24	20
Zinc	20	15
Copper	2	
Beta-carotene		800
Lutein	10	
Zeaxanthin	2.6	
DHA	400	
Resveratrol	30	
Hydroxytyrosol	3	
Vitamin A		800
Manganese		2
Selenium		50 μg

*Unless otherwise indicated

Table S2. Adverse events.

**Intervention group**	**Control group**
Related to the eyes	*Related to the eyes*
Cataract progression (n=5)<br>Wet AMD (n=3)<br>Increased intraocular pressure (n=1)<br>Blood vessel rupture (n=1)<br>Postsurgical macular edema (n=1)<br>Vitreous hemorrhage (n=1)	Wet AMD (n=2)<br>Cataract development (n=1)<br>Ocular inflammatory process after intravitreal injection (n=1)<br>Sterile endophthalmitis (n=1)<br>Diffuse superficial keratitis (n=1)<br>Keratitis (n=1)
Unrelated to the eyes	*Unrelated to the eyes*
Heartburn (n=2)	Diarrhea and gastrointestinal discomfort (n=1)

Table S3. Changes in inflammatory markers and angiogenic factors at month 12.

	Intervention			Control			Mean difference (intervention-control)	p-value	95% CI		Effect Size
**Variable**	**N**	**Mean change**	**SD**	**N**	**Mean change**	**SD**		**Student's t-test**	**Lower**	**Upper**	**Cohen's d**
**CYTOKINES (pg/mL)**											
**IL-8**	41	-0.8	3.49	45	1.64	6.44	-2.44	0.031	-4.64	-0.23	0.47
**IL-1β**	32	-0.58*	1	31	0.059	1.43	-0.63	0.045	-1.25	-0.014	0.52
**IFN-ɣ**	42	-2.83**	4.1	45	-1.3	6.6	-1.53	0.200	-3.89	0.82	0.28
**IL-9**	41	0.02	7.7	45	1.44	11.72	-1.42	0.513	-5.72	2.88	0.14
**MCP1**	42	-5.75	31.03	46	0.88	47.65	-6.62	0.447	-23.84	10.6	0.16
**TNF-α**	42	-2.46*	6.38	45	0.83	6.25	-3.28	0.017	-5.98	-0.59	0.52
**IL-10**	41	-1.32	4.85	43	-0.06	5.77	-1.26	0.282	-3.58	1.06	0.24
**IL-6**	34	-0.71	4.21	35	1.99	7.47	-2.7	0.070	-5.63	0.22	0.44
**IL-12p70**	34	-1.19	3.48	33	-0.81	3.57	-0.38	0.662	-2.1	1.34	0.11
**METALLOPROTEINASE**											
**MMP-10**	40	-17.31*	40.28	43	-20.3	83.85	2.99	0.835	-25.57	31.55	-0.05
**ANGIOGENIC FACTORS**											
**VEGF**	43	-47766.78	467858.99	46	-28940.55	509569.43	-18826.23	0.857	-225364.09	187711.63	0.04

CI, confidence interval; IFN, interferon; IL, interleukin; MCP, monocyte chemoattractant protein; MMP, matrix metalloproteinase; N, number of evaluable patients; TNF, tumor necrosis factor; VEGF, vascular endothelial growth factor

*p<0.01 and **p<0.001 vs. baseline

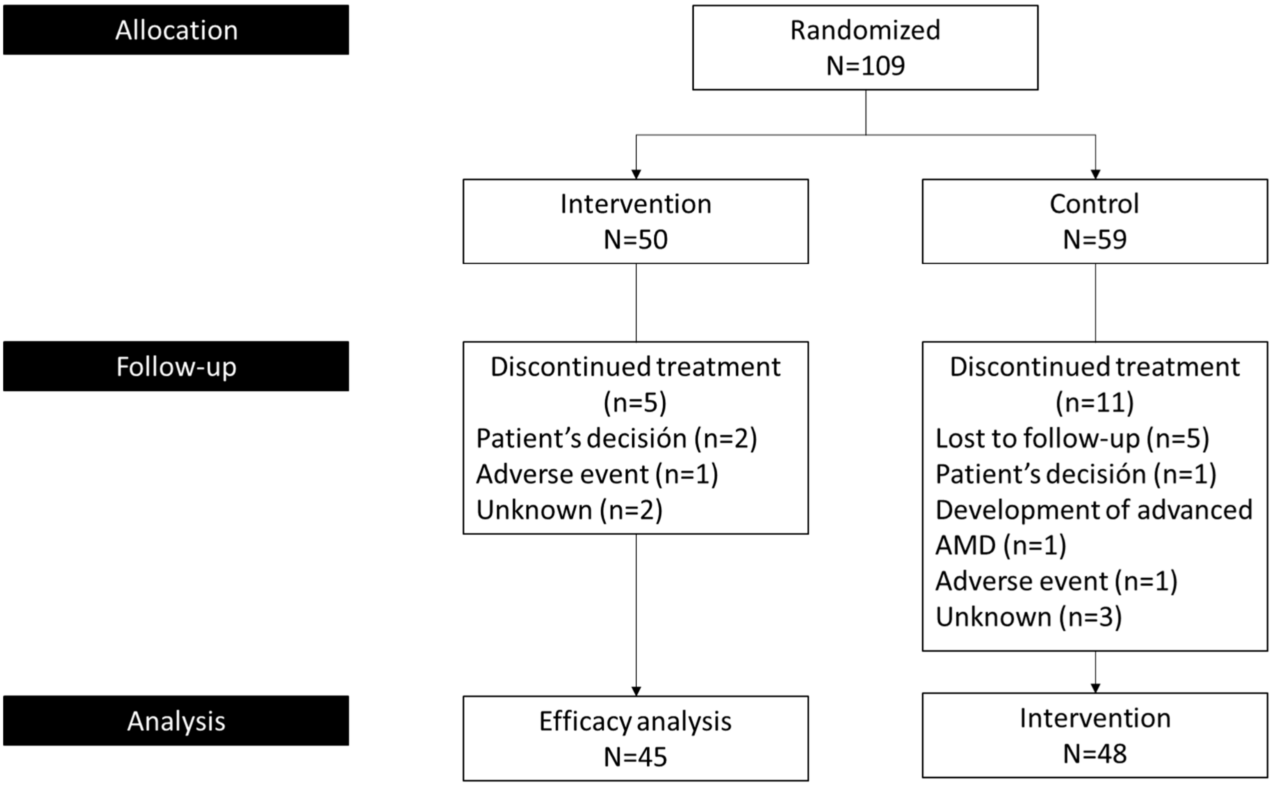

Figure S1. Patient disposition.
